# The Dutch Implantable Cardioverter–Defibrillator Decision Aid in Clinical Practice: A Stepped-Wedge Randomized Controlled Trial

**DOI:** 10.1177/0272989X261438122

**Published:** 2026-06-07

**Authors:** Dilek Yilmaz, Anastasia D. Egorova, Robert Grauss, Han A. M. Spierenburg, Kevin Venooy, Leon P. M. van Woerkens, Ramon Robles de Medina, Martin J. Schalij, Lieselot van Erven

**Affiliations:** Leiden University Medical Center, Leiden, The Netherlands; Leiden University Medical Center, Leiden, The Netherlands; Haaglanden Medical Center, The Hague, The Netherlands; Sint Maarten Medical Center, Cay Hill, Sint Maarten; Maastricht University Medical Center, Maastricht, The Netherlands; Albert Schweitzer Hospital, Dordrecht, The Netherlands; Haga Hospital, The Hague, The Netherlands; Leiden University Medical Center, Leiden, The Netherlands; Leiden University Medical Center, Leiden, The Netherlands

**Keywords:** implantable cardioverter–defibrillator, shared decision making, decision aid, randomized controlled trial, pulse-generator replacement

## Abstract

**Introduction:**

The role of shared decision making (SDM) has become increasingly pivotal, particularly in nuanced choices such as those involving implantable cardioverter–defibrillator (ICD) therapy. This study evaluates the impact of the Dutch ICD Decision Aid on SDM in patients up for ICD implantation or replacement.

**Methods:**

A stepped-wedge randomized controlled trial was conducted across 6 Dutch hospitals between February 2018 and September 2019, involving patients eligible for ICD implantation or pulse-generator exchange. SDM experiences of the patients and involved medical professionals were assessed using SDM-Q-9 and SDM-Q-Doc questionnaires, respectively. The Decisional Conflict Scale (DCS) scores measured effective decision making. The intervention group received the decision aid on top of standard care.

**Results:**

A total of 150 patients and 233 health care providers were included in the study. For health care providers, SDM scores did not differ: the SDM-Q-Doc median score was 36 (28–38) in the control phase and 35 (33–40) in the intervention phase (*P* = 0.81). Patients in both the intervention and control groups demonstrated high SDM scores as well. Decisional conflict scores were low: the median DCS score was 12.5 (4.3–23.4) in the intervention phase and 16.4 (6.25–25.0) in the control phase (*P* = 0.45). Patients with a higher education provided more correct answers to the theoretical knowledge questions. In addition, patients up for a pulse-generator exchange also had significantly more correct answers.

**Conclusions:**

Although the Dutch ICD Decision Aid did not result in significant differences in SDM scores or levels of decisional conflict between patient groups, both measures remained consistently favorable overall. The decision aid still holds promise as a valuable resource. Efforts should focus on refining decision-making tools and improving patient knowledge and the quality of patient-centered care.

**Highlights:**

- A digital decision aid did not significantly increase shared decision-making (SDM) scores for patients and health care providers, as SDM levels were already high across all groups.
- Despite high SDM scores, patient knowledge about implantable cardioverter–defibrillator (ICD) therapy remained low, highlighting a gap in understanding.
- Patients with higher education or prior experience with ICDs demonstrated better knowledge retention, indicating the need for tailored educational interventions.
- The study emphasizes the ongoing challenge of ensuring unbiased, well-informed decision making in ICD therapy, especially during pulse-generator replacements.

## Graphical abstract

**Graphical abstract:**
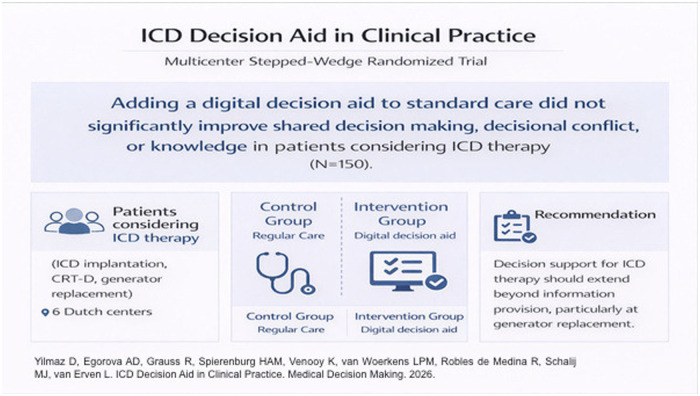

In recent years, shared decision making (SDM) has been established as an essential component of patient education in health care–related choices.^1,2^ Governmental campaigns in Europe and around the world have promoted the use of SDM modalities, with support from various decision aids to enhance the process.^3,4^ SDM is of particular value in patients facing treatment choices that require an individualized weighting of several complex factors and can significantly affect their quality of life.^5^

Implantable cardioverter–defibrillator (ICD) patients present a diverse and complex population within cardiology. While current guidelines recommend ICD implantation for selected patient populations, individual nuances related to the patient specific risks, comorbidity, preferences, and life perspectives can significantly affect treatment decisions.^6–9^ This is especially true for patients who previously underwent an ICD implantation and are now facing battery depletion–driven generator replacement. Patients may have developed significant comorbidities, and important changes in their life values and preferences may have occurred, which can affect their present-day treatment choices.^6^ Previously, patients reported not to have been fully involved in the decisional processes preceding the initial ICD implantation.^10^

To support patients and their health care providers in the SDM process concerning treatment with ICDs within the Netherlands, the online-based Dutch ICD Decision Aid was developed.^10,11^

Building on prior studies such as the DECIDE-ICD trial^12,13^ and other regional initiatives^14^ that demonstrated the relevance and feasibility of SDM in ICD patient populations, we aimed to evaluate the real-world implementation of a decision aid in the Dutch health care context, using a multicenter stepped-wedge randomized controlled trial.

## Methods

### Study Design

A stepped-wedge randomized controlled trial recruiting participants from 6 ICD-implanting hospitals in the Netherlands (Leiden University Medical Center, Leiden [1]; Haaglanden Medical Center, the Hague [2]; HagaZiekenhuis, the Hague [3]; St. Franciscus Gasthuis, Schiedam [4]; Albert Schweitzer Hospital, Dordrecht [5]; and Maastricht University Medical Center, Maastricht [6]) was conducted between February 2018 and September 2019.

**Figure 1:**
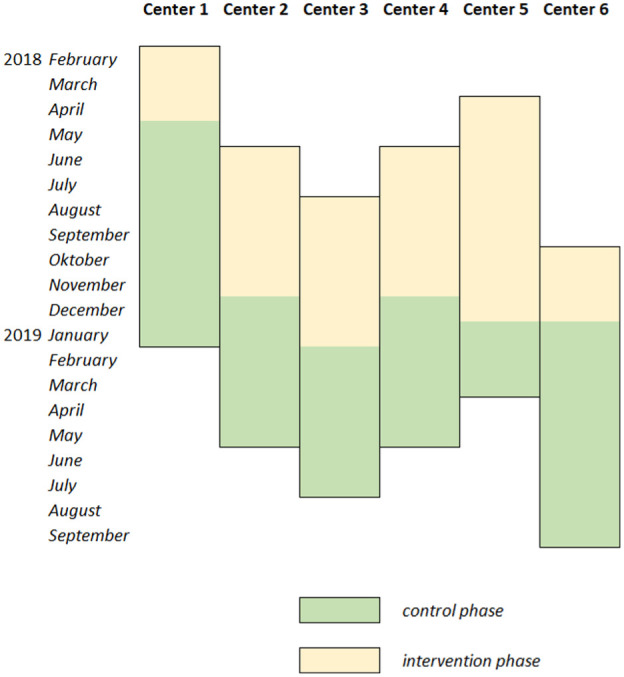
Schematic representation of the stepped-wedge cluster randomized trial design and the study phases distribution for participating hospitals 1 to 6. The control phase in shown in yellow: standard care enrollment period. The intervention phase is in green: decision aid implementation on top of the standard care enrollment period.

**Figure 2:**
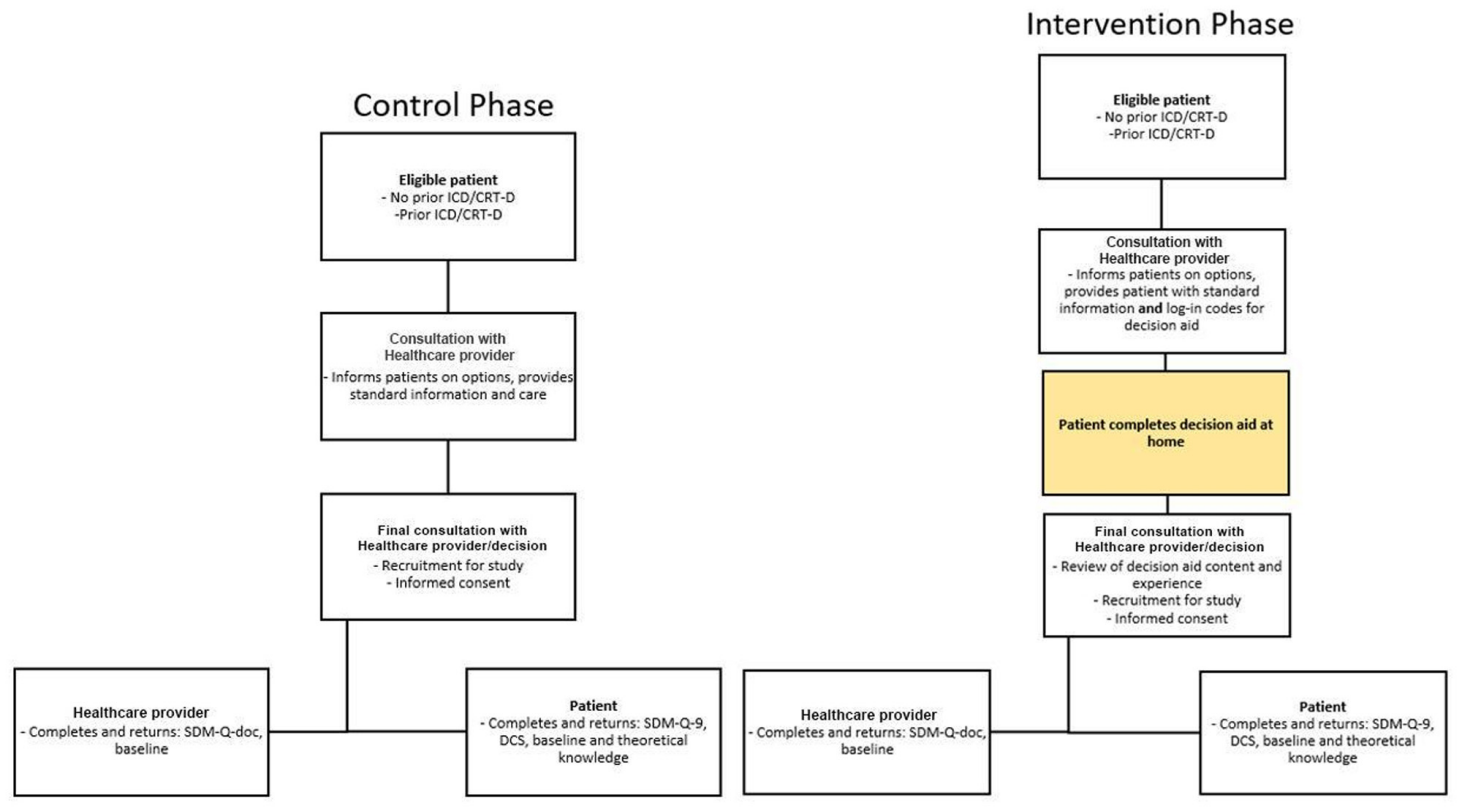
Study workflow. Schematic overview of the study procedures during the control and intervention phases. Patients received standard counseling alone or standard counseling plus the decision aid and completed study questionnaires together with their health care providers.

The trial design was in accordance with the stepped-wedge cluster randomized controlled trial (RCT) consensus, the Patient Decision Aid Standards, and the RAND-UCLA/multistepped Delphi model.^15–17^ Hospitals 1 through 6 were randomly assigned to either a variable duration of standard of care (control group) or the decision aid implementation on top of standard of care (intervention group). All of the centers initially enrolled patients into the control phase, and subsequently, each of the centers transitioned from the control to the intervention phase at a different point in time, as per study design (Figure 1). The periods of time for each group were 3 mo in the control phase and 9 mo in the intervention phase, 6 mo in the control phase and 6 mo in the intervention phase, and 9 mo in the control phase and 3 mo in the intervention phase, respectively. Figure 2 provides a schematic overview of the study design.

### Randomization

Prior to the start of the study, it was determined that a center could enroll in the control phase for 25%, 50%, of 75% of the total study enrolling period, after which the intervention phase time period would start. The primary investigator (D.Y.) randomized the centers using an online-based randomization tool to the previously specified enrollment patterns. Due to the nature of the study, blinding of participants and investigators was not possible.

### Participants

Patients aged 18 y or older who had an indication for an ICD or implantable cardiac resynchronization therapy–defibrillator (CRT-D) implantation or pulse-generator exchange due to battery depletion according to the European Society of Cardiology (ESC) guidelines were included.^18^ Patients with insufficient proficiency in the Dutch language were excluded from participation in the study.

In the standard care phase (control phase), patients were asked to fill out the study questionnaires to assess the consultation with their health care provider in which the ICD/CRT-D was discussed as a treatment option, the level of experienced SDM, and their decision-making progress (i.e., the experience, decisional conflict). In addition, 3 relevant theoretical questions were included to evaluate patient knowledge at the end of the decision-making process. Patients returned the questionnaires by post to the primary investigator (D.Y.). In the phase with the addition of the decision aid (intervention phase), participating hospitals provided all eligible patients with a decision aid as part of their standard practice. Patients in this phase received personal and unique codes from their health care providers to log into the online ICD Decision Aid environment. Patients reviewed the Decision Aid at home and had a follow-up visit with their health care provider to discuss the outcome and their final decision. Patients were asked for inclusion in the study and received the same questionnaires as the control group.

In the Netherlands, multiple health care providers are involved in the care of ICD patients prior to an ICD implant, including cardiologists and nurse practitioners or physician assistants (PAs). In all patients, the indication for an ICD implantation or pulse-generator exchange due to battery depletion was assessed by a cardiologist in accordance with the ESC guidelines.^18^ In the Dutch setting, both general and implanting cardiologists or electrophysiologists may lead ICD counseling, depending on local practice and individual patient care pathways. Implantation itself is performed exclusively by implanting cardiologists or electrophysiologists.

Patients were then individually counseled on their options by either the nurse practitioner/PA or the cardiologist, according to the hospitals’ local practice. These health care providers were asked to fill in the health care provider–specific study questionnaires throughout the study (during the control and the intervention phase). Besides their personal demographic characteristics, the health care providers filled out the modified SDM-Q-Doc.^19,20^ Based on unique inclusion numbers, health care provider questionnaires could be matched with patient questionnaires. Questionnaires in the first phase were filled out after the standard patient consultation. In the decision aid phase, questionnaires were filled out and returned after evaluating the decision aid with the patient and its outcome.

### Questionnaires

To assess the effectiveness of the decision aid, a survey was completed by patients and physicians after the final consultation in which a final decision for therapy was made. Both the patient and the physician questionnaire consisted of the 9-item SDM questionnaire, which measures the extent to which patients are involved in the process of decision making during a consultation from the perspective of the patient (version SDM-Q-9) and the perspective of the physician (SDM-Q-Doc).^19,20^ The SDM-Q, a self-reported survey, consists of 9 items that each describe a different step of the SDM process and is the most frequently used instrument for assessing the involvement of the patient in medical decision making.^19^

In addition to the SDM-Q-9, the traditional 16-question Decisional Conflict Scale (DCS) was used for the patients to measure uncertainty in making a health-related decision, factors contributing to uncertainty and perceptions of effective decision making.^21^ The DCS expresses the amount of uncertainty within an individual. The DCS assesses aspects of decision making in 5 different subscales: 1) feeling uncertain about the best course of action, 2) feeling uninformed, 3) feeling unclear about values, 4) experienced support during the decision-making process, and 5) ineffective decision making. The items are measured on a 5-point Likert scale (0 = *strongly agree* to 4 = *strongly disagree*), which gives a total score ranging from 0 (no decisional conflict) to 100 (extremely high decisional conflict). For all subscales, a higher score indicates greater decisional conflict and thus experiencing more uncertainty and less effective choices. The decisional conflict score was determined according to the user manual.^21^ A total score of 25 or less is associated with no decisional conflict. A total score greater than 25 is associated with decisional conflict, and scores higher than 37.5 are specifically associated with decision delay or feeling clearly unsure about implementation.^21,22^ In addition to the questionnaires, patients’ knowledge on the ICD/CRT-D was tested using 4 knowledge questions regarding the ICD/CRT-D implantation and risks. For the analyses, the statements were dichotomized: correct or incorrect answer or missing answer.

Data on patients logged on to the online decision aid, including number of logged-in sessions and the duration in time spent online, were collected retrospectively from the online environment log file.

For the English translation of the study questionnaires, see Appendix 1 and 2.

### Primary Outcomes

The primary outcome of this study was the degree of SDM experienced by patients and health care providers with or without the decision aid implementation (based on the SDM-Q-9 and SDM-Q-doc). The additional primary outcome for patients was the experienced decisional conflict.

### Secondary Outcomes

Knowledge was assessed by the 4 theoretical questionnaires.

### Data Collection

In case of 1 or 2 missing answers in the SDM-Q-Doc, the mean of the available answers was inputted to the missing answers. Questionnaires missing more than 2 answers were excluded from analysis. All items are scored on a 6-point Likert scale. Scores were aggregated to a raw score between 0 and 45, with 0 indication the lowest and 45 the highest level of SDM. Following Kriston et al.,^23^ the score was transformed by multiplication by 20/9 to get a score range from 0 to 100, as this range is intuitively better interpretable, where 0 represents the lowest level and 100 the highest level of SDM. Higher scores on SDM-Q-9 and SDM-Q-Doc indicate greater SDM (range 0–100). Lower scores on the DCS indicate less conflict (range 0–100). Characteristics of the total score were analyzed, and the distributions were checked. Differences in total score between the control group and the decision aid group were analyzed using linear mixed modeling. For the SDM-Q-9 questionnaires for the patients, the same methods were used as for the SDM-Q-Doc questionnaires.

In case of the SDM questionnaires (SDM-Q-9 and SDM-Q-doc), missing answers to more than 2 questions were excluded from the analysis. In the case of 1 or 2 missing values, these were corrected by imputation: the imputed score was the mean score of the present variables.^10,19^ In case of missing answers in the decisional conflict questions, multiple imputation was used.

For the 4 questions on patients’ theoretical knowledge, all correct answers provided were added up per patient, and the score was expressed as percentage correct answers of the total number of 4 questions (i.e. 0%, 25%, 50%, 75%, or 100% correct).

### Statistical Analyses

We conducted both an intention-to-treat (ITT) and a treated-as-intended (TAI) analysis. The ITT analysis included all patients according to the stepped-wedge cluster allocation, irrespective of actual use of the decision aid. The TAI analysis included only patients for whom the decision aid was confirmed to have been used during the consultation, based on clinician self-report. This allowed us to explore potential differences related to the actual implementation of the intervention.

On the basis of the normality of distribution, continuous variables are presented as mean ± SD or median with interquartile range (IQR) (25th to 75th percentile). All outcomes were analyzed as intention to treat and as treated.^17,24^ The differences between the control and the intervention group in total DSC score patients and in SDM scores for patients and health care providers were evaluated using linear mixed modeling. The differences in DC subscores were analyzed using linear mixed modeling analysis. Differences in the categorical values were analyzed using χ^2^ square test. A *P* value of >0.05 was considered statistically significant.

Results in the tables and text are presented for both ITT and TAI analyses to reflect allocation versus actual use of the decision aid.

### Ethics Statement

The study was conducted in accordance with the Declaration of Helsinki, applicable local laws and regulations, and the European directive for data protection (General Data Protection Regulation).

The Medical Ethical Committee of the Leiden University Medical Center approved the study protocol (P16.096). This was endorsed by the local ethical committees of all the individual participating centers. Each patient provided written informed consent for participation in the study.

## Results

**Figure 3:**
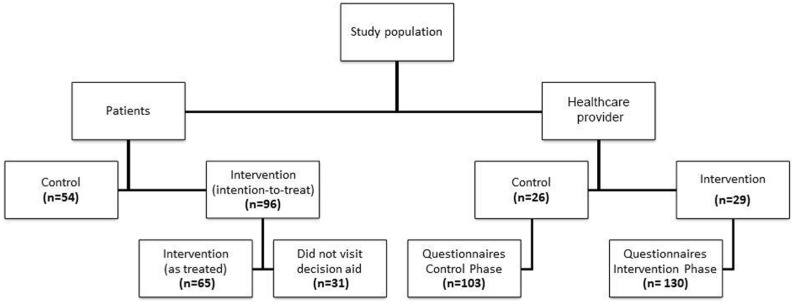
Study population. Flow diagram of patient and health care provider inclusion. Patients were enrolled during the control and intervention phases, and treated-as-intended analyses included only patients who accessed the decision aid.

A total of 150 patients were included in the study. Figure 3 shows the schematic representation of the study population and inclusions.

The control phase included 54 patients, and the intervention phase included 96 patients. Of all patients, 34 (23%) were female, the median age was 68 (59–77) y, and 64 (43%) patients were up for a pulse-generator exchange. There was no difference between the study groups for these baseline characteristics. Furthermore, there were no differences in level of education between the groups (i.e., proportions of higher v. lower education; Table 1). Of the total study population, 97 patients (65%) underwent an ICD implantation (ventricular rate or dual rate system), and 53 patients (35%) underwent a CRT-D implantation. This distribution did not differ significantly between the control and intervention phases (Table 1B; *P* < 0.05). In total, 65 (68%) were confirmed to have logged into the decision aid online platform. Most of these patients from the treated-as-intended arm logged in once (72%) onto the decision aid. The median time spent online on the decision aid was 16 (8.75–50.0) min (Supplemental Table 5).

**Table 1:** Baseline Patient Characteristics

	Control Phase (n = 54)	Intervention Phase (n = 96)	Treated as Intended (n = 65)	Intention to Treat v. Control, P Value	Treated as Intended v. Control, P Value
Median age, y (IQR)	69 (60–78)	67 (58–75)	69 (63–76)	0.68	0.82
Female sex, n (%)	15 (18)	27 (19)	20 (31)	0.74	0.92
Up for pulse-generator exchange, n (%)	16 (30)	48 (50)	30 (46)	0.55	0.016
Refrained from ICD implantation/ replacement, n (%)	2 (0.04)	0 (0)	0 (0)	0.93	1.00
Type of device, n (%)^a^				0.99	0.90
ICD	34 (64)	63 (66)	41 (63)		
CRT-D	20 (36)	33 (34)	24 (37)		
Educational level, n (%)				0.99	0.94
Elementary school	4 (7)	9 (9)	5 (8)		
Lower vocational	8 (17)	12 (13)	5 (8)		
Lower secondary	7 (13)	11 (11)	5 (11)		
Intermediate vocational	13 (24)	27 (28)	19 (29)		
Higher secondary	4 (7)	6 (6)	5 (9)		
Higher vocational	12 (22)	18 (19)	14 (22)		
University	0 (0)	9 (9)	5 (11)		
Unknown/other	6 (11)	2 (2)	2 (3)		

CRT-D, cardiac resynchronization therapy; ICD, implantable cardioverter–defibrillator; IQR, interquartile range (25th–75th percentile).

a The device type for which the patient was a candidate.

**Figure 4:**
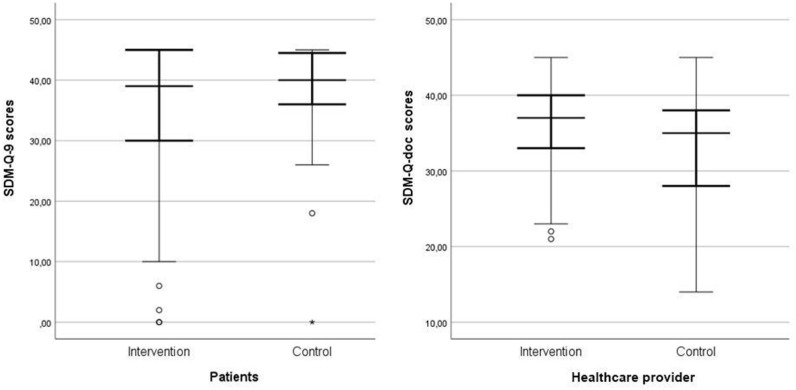
Shared decision-making scores. Boxplots showing shared decision-making scores for patients (SDM-Q-9) and health care providers (SDM-QDoc) during the control and intervention phases. The median values and interquartile ranges are displayed.

The baseline characteristics of the treated-as-intended group did not differ from the controls or intention-to-treat group, except for a difference in number of pulse-generator exchange patients (Table 1). SDM scores were overall high, and the experienced decisional conflict was low (Figure 4 and Table 3). The median SDM-Q-9 score for patients was 39 (36–45) in the control phase and 40 (30–45) in the intervention phase (*P* = 0.25). DC scores were low: the median DCS score for patients was 16.4 (6.25–25.0) in the control phase and 12.5 (4.3– 3.4) in the intervention phase (*P* = 0.45).

A total of 233 health care provider questionnaires were included for analysis, filled out by 26 unique health care providers in the control phase and 29 unique health care providers in the intervention phase. Each questionnaire completed by a health care provider corresponds to a unique patient encounter. For example, if a provider completed 4 questionnaires, these reflect 4 separate patients for whom they provided ICD counseling. No repeated measures were collected for the same patient over time. Of these unique health care providers, 9 (69%) were female. The median age was 42 (36–48) y, and the median clinical experience was 17 (12–22) y. There were no differences between the groups in baseline characteristics (Table 2). The SDM scores were overall high. There was no difference between the 2 phases for the group of health care providers. The median SDM-Q-Doc score was 36 (28–38) in the control phase and 35 (33–40) in the intervention phase (*P* = 0.81; Figure 4).

**Table 2:** Baseline Characteristics of the Health Care Providers

	Control Phase	Intervention Phase	P Value
Total questionnaires	103	130	
Total unique health care providers	26	29	0.82
Cardiologist	8 (31%)	9 (31%)	0.97
Nurse practitioner	18 (69%)	20 (69%)	0.43
Female sex	19 (73%)	66 (34%)	0.51
Age, y, median (IQR)	43 (38–48)	40 (35–48)	0.54
Median years of clinical experience (IQR)	18 (15–22)	17 (12–22)	0.50

IQR, interquartile range (25th and 75th percentile). Each completed questionnaire represents a unique patient; no repeated measures per patient were collected.

For patients, with both the intention-to-treat and treated-as-intended analyses, there were no differences in decisional conflict or SDM experience, also not when corrected for type of health care provider, patient age, patient gender, or patient’s level of education (Table 2B).

The theoretical knowledge as assessed by the 4 knowledge questions was overall low. The response rate was 150/150 (100%). Two or more correct answers were provided by 29 (54%) patients in the control group and 58 (60%) patients in the intervention group (*P* = 0.146; Supplemental Table 2).

Patients with a higher education provided more correct answers to the theoretical knowledge questions, in both the control and the intervention/decision aid phase (*P* < 0.001). In addition, patients up for a pulse-generator exchange also had significantly more correct answers, compared with peers up for a first device implantation, in both the control and the intervention/decision aid phase (*P* < 0.001; Supplemental Table 3).

## Discussion

SDM has gained prominence in health care, emphasizing the importance of involving patients in treatment decisions to align care with their values and preferences. The current study focused on the unique context of ICD patients, a population with complex considerations in treatment choices. Our findings should be interpreted in the context of prior studies assessing decisional conflict and SDM in ICD patients.^12,13,25^ Multiple trials have evaluated these outcomes, including in advanced cardiology populations, highlighting the relevance and growing attention to SDM in this field. Our study adds to this literature by providing data from a European cohort, where cultural and health care system differences may influence decision-making processes. These contextual factors are important when comparing findings across studies.

The main finding of this multicenter stepped-wedge randomized controlled trial is that in the current Dutch practice, patients as well as health care providers experienced high levels of SDM, as reflected by high SDM scores reported by both patients and health care providers, regardless of decision aid implementation and actual utilization. This decisional conflict, measured by the DCS, was also low for all patient groups. Nevertheless, the theoretical knowledge as assessed by the 4 knowledge questions was overall low. Patients with a higher education and those with experience provided more correct answers to the theoretical knowledge questions.

Although the results reported here are consistent with high patient satisfaction levels in the Netherlands in general,^26^ they stand in remarkable contrast to a previous study on Dutch ICD patients, which retrospectively reported low levels of SDM.^10^ This could have several reasons. First, the design of that study was prone to recall bias, as patients were surveyed in a retrospective manner. In addition, it was reported that only a minority of Dutch clinics invite their patients for a consultation and exploration of options when they are up for a pulse-generator exchange at battery depletion.^27^ In light of the contrast with these previous findings, it can be considered that the adjustments made to patients’ education and standard care during or prior to the initiation of the trial may have favorably biased the study outcomes, a phenomenon recognized as the “Hawthorne effect.”^28,29^

Furthermore, while decisional conflict was low and SDM scores were high, the decision aid has value beyond being an informational tool. By structuring the consultation and prompting reflection on personal values, the aid supports patients in moving from passive information receipt to active participation in decision making. Thus, it has potential to reinforce SDM rather than function as a 1-directional educational tool only.

Another contextual factor worth noting is the relatively young age yet considerable clinical experience of the participating health care professionals. It is conceivable that this demographic profile reflects a generation of providers more accustomed to patient-centered care principles and less inclined toward a paternalistic approach. This may partly explain why the additional introduction of the decision aid did not lead to a measurable change in their reported SDM behavior.

Furthermore, it has previously been described that doctors have a leading role in decision making for patients eligible for an ICD.^30^ Strong language emphasizing the benefits of an ICD is likely to lead to patients favoring device implantation or replacement. Previous interviews with patients repeatedly highlighted cognitive biases in ICD patients favoring ICD therapy.^31^ Also in the aforementioned study, patients reported to be influenced by counseling with favorable framing of the ICD by their physicians. These findings reaffirm the necessity for an unbiased decision aid and underlines that the comparable SDM levels in all groups of this study may not be a reliable representation of true clinical practice.

Moreover, in our study, despite high scores on SDM, objective scores on knowledge overall were low, suggesting that patients may in fact be unconsciously uninformed. This highlights the importance of providing patients with adequate information and resources to make well-informed decisions.

In addition, among the study patients provided with log-in codes for the decision aid, only 65% accessed this information. However, even though this participation rate may seem modest, it is comparable to or even exceeds response rates reported in similar studies, such as the one conducted by Etnel et al.^32^ in patients with congenital aortic and pulmonary valve disease, with only 51% of subjects in the intervention group actually visiting the information portal. The challenges associated with online patient engagement are therefore not unique to this specific decision aid.

### Misconceptions and Information Comprehension

Traditionally, the pros and cons of treatment options are either summed up or illustrated by percentages. The comprehension of patients of mere percentages has been shown to be disappointing.^33^ Moreover, it has been previously established that ICD patients overestimate benefit from ICD therapy and are deficient in their comprehension of device function.^34–38^ Aside from benefit, ICD patients have previously reported to have not understood fully the risks and burden of living with an ICD at time of ICD implantation.^30,34^ On the contrary, patients who had previously declined an ICD implantation for primary prevention had not fully understood the benefits for survival.^39^

Patients nevertheless desire to have access to comprehensive information that can help them in making a decision. Providing patients with comprehensive information and taking into account their preferences are important for sustainable decision making. Interestingly, traditional print-based educational material for ICD patients have previously be proven to be targeted at a highly literate population.^40^ For this reason, the decision aid in this study was available online, interactive, and incorporated illustrative educational videos in simple language. Due to the potential limitation of digital illiteracy, the content of the decision aid was also available for printing by health care providers for their patients. These patients were, however, not eligible for study inclusion. In addition, all text was reviewed by professional content writers to be comprehendible for the lower-literate population.^11^

### Previous ICD Decision Aids

Previously, with 18 participants in total, Lewis et al.^41^ developed a user-centered ICD decision aid that was positively evaluated by the participants to the study. The evaluation of the decision aid was based on interviews, and no trial for implementation in clinical practice was performed as in this study. Another study by Lewis et al.^42^ focused on 30 patients who were randomized to a decision support intervention (included a paper-based patient decision aid and nurse-led coaching) before ICD–battery exchange procedure. The authors concluded that the decision support intervention had “the potential to improve ICD replacement decision quality,” based on improved knowledge outcomes in this small patient population. No standardized scores were used in the evaluation, and patient numbers were small.

Our findings complement prior US-based trials of ICD decision aids, but cultural and systemic differences in health care delivery may partly explain differences in observed outcomes. The Dutch context, with a strong tradition of patient involvement and universal health care coverage, may limit the generalizability of our results to other settings.

In light of these studies, our trial provides insights from a larger study group, with a digital decision aid implemented and evaluated in a RCT setting, using validated questionnaires as a study outcome.

In line with recent insights into the evolving role of patient decision aids, sustained implementation and regular updating of tools are essential to effectively embed SDM into clinical practice.^43^ To support long-term sustainability, the decision aid was developed in collaboration with ZorgKeuzeLab, an organization responsible for maintenance, technical support, and promotion. In addition, funding from the Netherlands Heart Registration ensures that patients can access the tool free of charge, even if their hospital has not formally adopted it.

### Decision Making at Time of Battery Depletion

Interestingly, patients up for a pulse-generator exchange accessed the decision aid more frequently than their de novo peers did. This insight was provided by the treated-as-intended analysis of our study, for the baseline of the 2 groups. Notably, patients undergoing generator replacement also performed better on knowledge questions compared with the novo implants. However, the fact that relatively basic questions were still answered incorrectly suggests that living with an ICD does not automatically ensure a complete or up-to-date understanding of the therapy. This highlights a potential blind spot, in which patients may be unaware of knowledge gaps, also referred to as”‘unconsciously uninformed.” These findings underscore the value of structured counseling during generator replacement consultations, not only to refresh key information but also to reassess whether continued ICD therapy aligns with patients’ current health status and personal preferences. Yet, our previous work^27^ demonstrated that at time of ICD replacement, this was not always considered a choice by health care providers. Likewise, it has been previously shown that more than half of the patients who had already undergone an ICD replacement at time of battery depletion were not aware that they had a choice.^27,44^ This illustrated that ICD replacement at time of battery depletion in real-life practice often goes without saying, whereas patients have been reported to consider nonreplacement under certain circumstances such as serious illness and advanced age.^44^

Moreover, patient preferences can change with the progression of age and the involvement of new comorbidity. An ICD decision aid can also facilitate decision making in these patients, exploring their current preferences and weighing them out against expected benefits and downsides from ICD therapy.

The inclusion of a heterogeneous patient cohort, encompassing individuals considered for a first ICD implantation, CRT-D implantation, or generator replacement in this study, reflects real-world clinical practice, where SDM must address a broad spectrum of clinical scenarios and patient trajectories. While this approach enhances the external validity and generalizability of our findings, we recognize that the different patient groups may experience distinct procedural risks, decision-making dynamics, and psychological considerations. For instance, patients undergoing generator replacement often face more complex reconsiderations after living with an ICD for several years, potentially with higher thresholds to withdraw from therapy. These nuances underscore the need for future studies to examine these subgroups in greater depth.

### Device Refusal

Interestingly, only 2 patients in this study ultimately declined ICD implantation. While this proportion aligns with the high acceptance rates seen in routine clinical practice for primary prevention ICDs, it may limit the ability to detect an effect of the decision aid on actual device refusal. This low refusal rate may partly reflect the inherent complexity of revisiting a prior ICD indication at generator replacement as well as the strong clinical guidance typically accompanying these decisions. It may also indicate that the decision aid did not clearly convey that declining or deferring ICD therapy is a medically reasonable option under certain circumstances. This represents an important opportunity for further refinement of the tool Unfortunately, no additional patient-level data were available to further explore the motivations or characteristics of these few patients.

### Limitations

This study has several limitations. First, previous reports of suboptimal patient involvement prior to generator exchange procedures may have raised awareness and improved standard care, potentially leading to a better-informed control group. The use of an online decision aid may have introduced selection bias due to digital literacy requirements, and patient surveys are inherently prone to recall bias.

The scope of our decision aid was limited to the choice of ICD implantation or replacement and did not address decisions involving CRT. As such, the choice between CRT-D and CRT-P was not included, and we did not collect specific data on CRT-D versus ICD-only implants. Future studies should account for this distinction.

Detailed clinical baseline characteristics—such as indication for primary versus secondary prevention or prior cardiovascular interventions—were not available due to ethical and privacy constraints. This limited our ability to link patient-reported outcomes to clinical context. Future research should aim to combine Patient Reported Outcome Measures (PROMs) with richer clinical data.

Patients undergoing ICD generator replacement were included without separate powering for this subgroup, which may have diluted the detectable effects. Stratified analyses in larger samples are warranted.

The heterogeneity of our cohort—combining ICD-only, CRT-D, and generator replacement patients—reflects real-world practice but may have contributed to the absence of clear differences in our primary outcomes. Future trials should be powered and designed to stratify by device type (and any concomitant pacing or resynchronization indication) to better understand the differential effects of decision aids in these distinct populations.

In addition, SDM was delivered by various providers, who may differ in communication style or familiarity with the decision aid. This variability may have affected consistency, although it also reflects typical multidisciplinary care pathways.

We did not assess consultation efficiency or long-term outcomes. Recall bias may have influenced patient-reported SDM scores. In our prior retrospective study, SDM levels were lower, possibly reflecting recall effects or differences due to the presence of a decision aid. Future studies could address this by collecting repeated measurements over time.

The wide confidence intervals around our estimates (Table 3) highlight the limited power to detect subgroup differences. This underscores that our null findings should be interpreted as inconclusive rather than as evidence that the intervention has no effect.

**Table 3:** Primary Outcomes in Patients

	Control Phase	Intervention Phase	Treated as Intended	Intention-to-Treat *P* Value	Treated-as-Intended *P* Value
SDM-Q-9, median (IQR)	39 (36–45)	40 (30–45)	39 (30–45)	0.180	0.122
DCS, median [IQR]	16.4 (6.25–25.0)	12.5 (4.3–23.4)	13.3 (4.7–23.4)	0.81	0.73
• Subscore uncertainty	25 (0–50)	16.6 (0–43.75)	16.7 (0–50)	0.54	0.63
• Subscore informed	8 (0–25)	0 (0–16.67)	0 (0–16.7)	0.63	0.60
• Subscore values	25 (0–33)	20.8 (0–33)	16.7 (0–33.3)	0.72	0.79
• Subscore support	16.6 (0–27)	16.6 (0–33)	8.3 (0–33.3)	0.53	0.97
• Subscore effective decision making	0 (0–12.5)	0 (0–6.25)	0 (0–6.25)	0.94	0.73
Chosen for no (longer) ICD therapy	2 (0.04%)	0 (0%)	0 (0%)	0.93	1.00

DCS, Decisional Conflict Scale score; ICD, implantable cardioverter–defibrillator; IQR, interquartile range (25th and 75th percentile); SDM-Q-9, shared decision-making scores based on the Shared Decision Making 9 questionnaire.

Despite these limitations, this is the largest randomized trial on SDM in ICD care to date, using validated tools for SDM and decisional conflict as key endpoints.

## Conclusion

This study assessed the impact of the Dutch ICD Decision Aid on SDM in the complex context of ICD patients facing the decision of a first device implantation or pulse battery depletion-driven generator replacement. Despite high SDM scores for both patients and health care providers, irrespective of decision aid utilization, knowledge levels remained suboptimal. The decision aid offers a valuable resource but highlights the ongoing need for refining tools to enhance unbiased decision making and improve patient knowledge in the complex landscape of ICD therapy. Future efforts should focus on addressing these challenges to promote informed and patient-centered decisions.

## Supplemental Material

sj-doc-1-mdm-10.1177_0272989X261438122 – Supplemental material for The Dutch Implantable Cardioverter–Defibrillator Decision Aid in Clinical Practice: A Stepped-Wedge Randomized Controlled Trial Supplemental material, sj-doc-1-mdm-10.1177_0272989X261438122 for The Dutch Implantable Cardioverter–Defibrillator Decision Aid in Clinical Practice: A Stepped-Wedge Randomized Controlled Trial by Dilek Yilmaz, Anastasia D. Egorova, Robert Grauss, Han A. M. Spierenburg, Kevin Venooy, Leon P. M. van Woerkens, Ramon Robles de Medina, Martin J. Schalij and Lieselot van Erven in Medical Decision Making

sj-docx-1-mdm-10.1177_0272989X261438122 – Supplemental material for The Dutch Implantable Cardioverter–Defibrillator Decision Aid in Clinical Practice: A Stepped-Wedge Randomized Controlled Trial Supplemental material, sj-docx-1-mdm-10.1177_0272989X261438122 for The Dutch Implantable Cardioverter–Defibrillator Decision Aid in Clinical Practice: A Stepped-Wedge Randomized Controlled Trial by Dilek Yilmaz, Anastasia D. Egorova, Robert Grauss, Han A. M. Spierenburg, Kevin Venooy, Leon P. M. van Woerkens, Ramon Robles de Medina, Martin J. Schalij and Lieselot van Erven in Medical Decision Making
