# Supplementary material for: The Dutch Implantable Cardioverter–Defibrillator Decision Aid in Clinical Practice: A Stepped-Wedge Randomized Controlled Trial
Source: Med Decis Making. 2026 Jun 7;46(6):744–56. doi: 10.1177/0272989X261438122 (PMC13346597; doi:10.1177/0272989X261438122)
Supplement: sj-docx-1-mdm-10.1177_0272989X261438122 – Supplemental material for The Dutch Implantable Cardioverter–Defibrillator Decision Aid in Clinical Practice: A Stepped-Wedge Randomized Controlled Trial [file sj-docx-1-mdm-10.1177_0272989X261438122.docx]

**Supplementary data**

|  | **Intervention phase** | **Treated as intended** | ***p-value*** |
| --- | --- | --- | --- |
| SDM-Q-9 | 40 [IQR 30-45] | 39 [30-45] | 0.995 |
| DCS | 12.5 [4.3 – 23.4] | 13.3 [4.7-23.4] | 0.256 |
| - *subscore uncertainty* | 16.6 [IQR 0 -43.75] | 16.7 [0-50] | 0.131 |
| - *subscore informed* | 0 [IQR 0 -16.67] | 0 [0-16.7] | 0.956 |
| - *subscore values* | 20.8 [IQR 0 -33] | 16.7 [0-33.3] | 0.334 |
| - *subscore support* | 16.6 [IQR 0 -33] | 8.3 [0-33.3] | 0.285 |
| - *subscore effective decision making* | 0 [IQR 0 -6.25] | 0 [0-6.25] | 0.851 |
| Chosen for no (longer) ICD therapy | 0 (0%) | 0 (0%) | n/a |

**Supplemental table 1:** primary outcomes*. SDM-Q-9: shared decision making scores based on the shared decision making 9 questionnaire. DCS: decisional conflict scale score. ICD: implantable cardioverter-defibrillator.*

|  | **Control phase** | **Intervention phase** | **Treated as intended** | | **Intention to treat vs control**  **p-value** | **Treated as intended p-value vs control** |  |
| --- | --- | --- | --- | --- | --- | --- | --- |
|  | (n= 54) | (n=96) | (n=65) | 0.146 | | 0.199 | |
| 0% correct | 3 | 4 | 3 |  | |  | |
| 25% correct | 0 | 6 | 4 |  | |  | |
| 50% correct | 9 | 17 | 10 |  | |  | |
| 75% correct | 20 | 21 | 15 |  | |  | |
| 100% correct | 22 | 47 | 33 |  | |  | |

**Supplemental table 2:** theoretical knowledge question results.

|  | **Control phase**  ***De novo*** | **Pulse-generator-exchange** | **Intervention phase**  ***De novo*** | | **Pulse-generator-exchange** | **Intention to treat vs control**  **p-value** |  |
| --- | --- | --- | --- | --- | --- | --- | --- |
|  | (n= 38) | (n=16) | (n=48) | (n=48) | | <0.001 | |
| 0% correct | 3 | 1 | 6 | 0 | |  | |
| 25% correct | 8 | 1 | 9 | 3 | |  | |
| 50% correct | 7 | 4 | 9 | 11 | |  | |
| 75% correct | 18 | 8 | 24 | 30 | |  | |
| 100% correct | 2 | 2 | 0 | 4 | |  | |

**Supplemental table 3:** theoretical knowledge question results, *de novo* patients versus patients for pulse-generator exchange.

|  | **Control phase** | **Intervention phase** | | | ***p-value*** | |
| --- | --- | --- | --- | --- | --- | --- |
| SDM-Q-Doc, median [IQR] | 36 [28-38 ] | | 35 [33 – 40] | 0.805 | |  |

**Supplemental table 4:** Primary outcome healthcare providers*. SDM-Q-doc: shared decision making scores based on the shared decision making doctor questionnaire. IQR: interquartile range* [*25^th^ and 75^th^ percentile*]*.*

|  | **Logged-in patients (n=65)** |
| --- | --- |
| Number of times logged-in |  |
| - *1* | 47 (72%) |
| - *2* | 7 (11%) |
| - *3* | 4 (6%) |
| - *4* | 7 (11%) |
| Median time online in minutes [IQR] | 16 [8.75 – 50.0] |

**Supplemental table 5:** treated-as intended arm patients logfile data. *IQR: interquartile range (25^th^ and 75^th^ percentile)*

**Link te decision aid (Dutch): <https://icd.keuzehulp.nl/>**
Screenshots of Decision Aid content


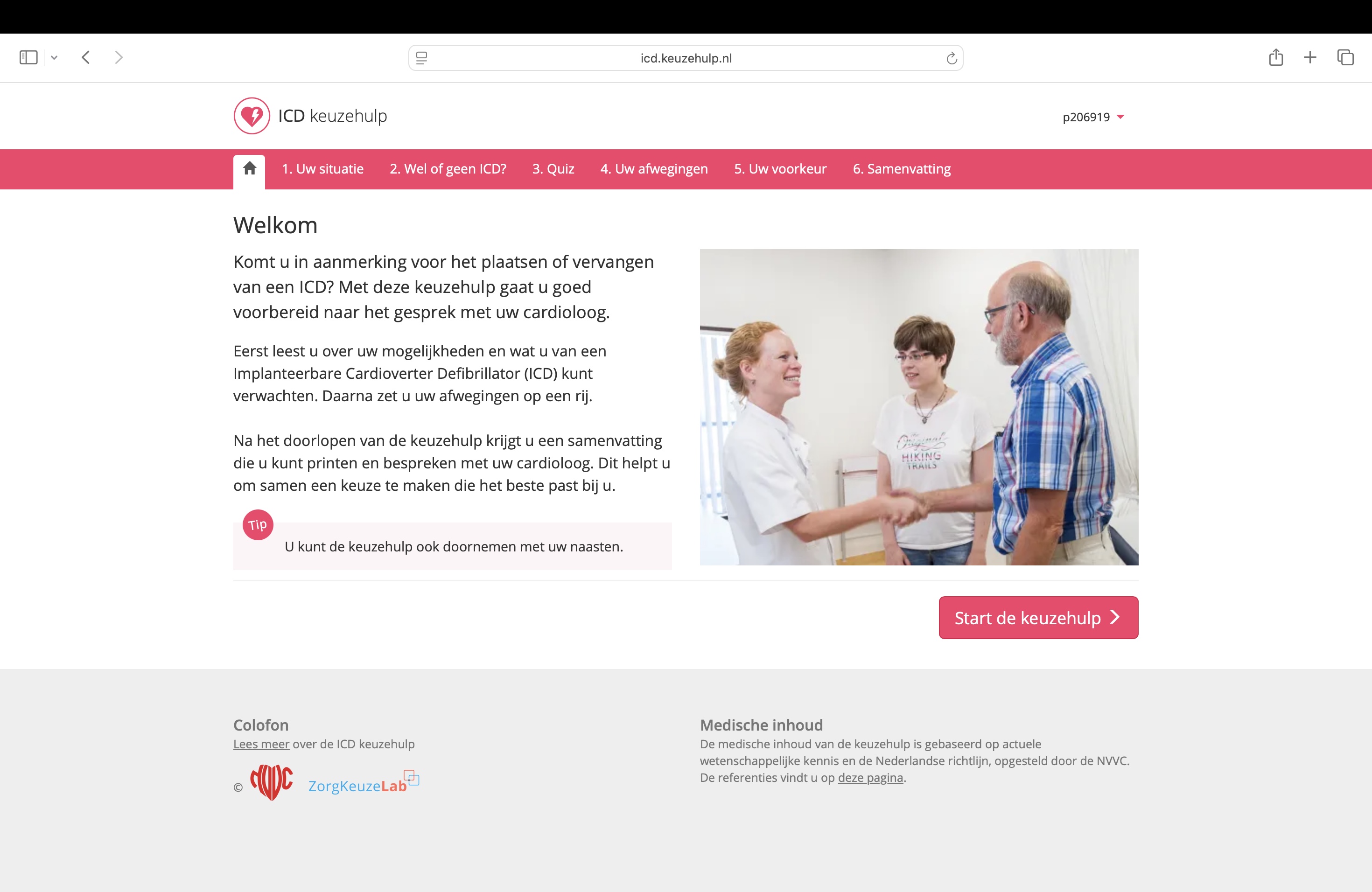


Introductory page


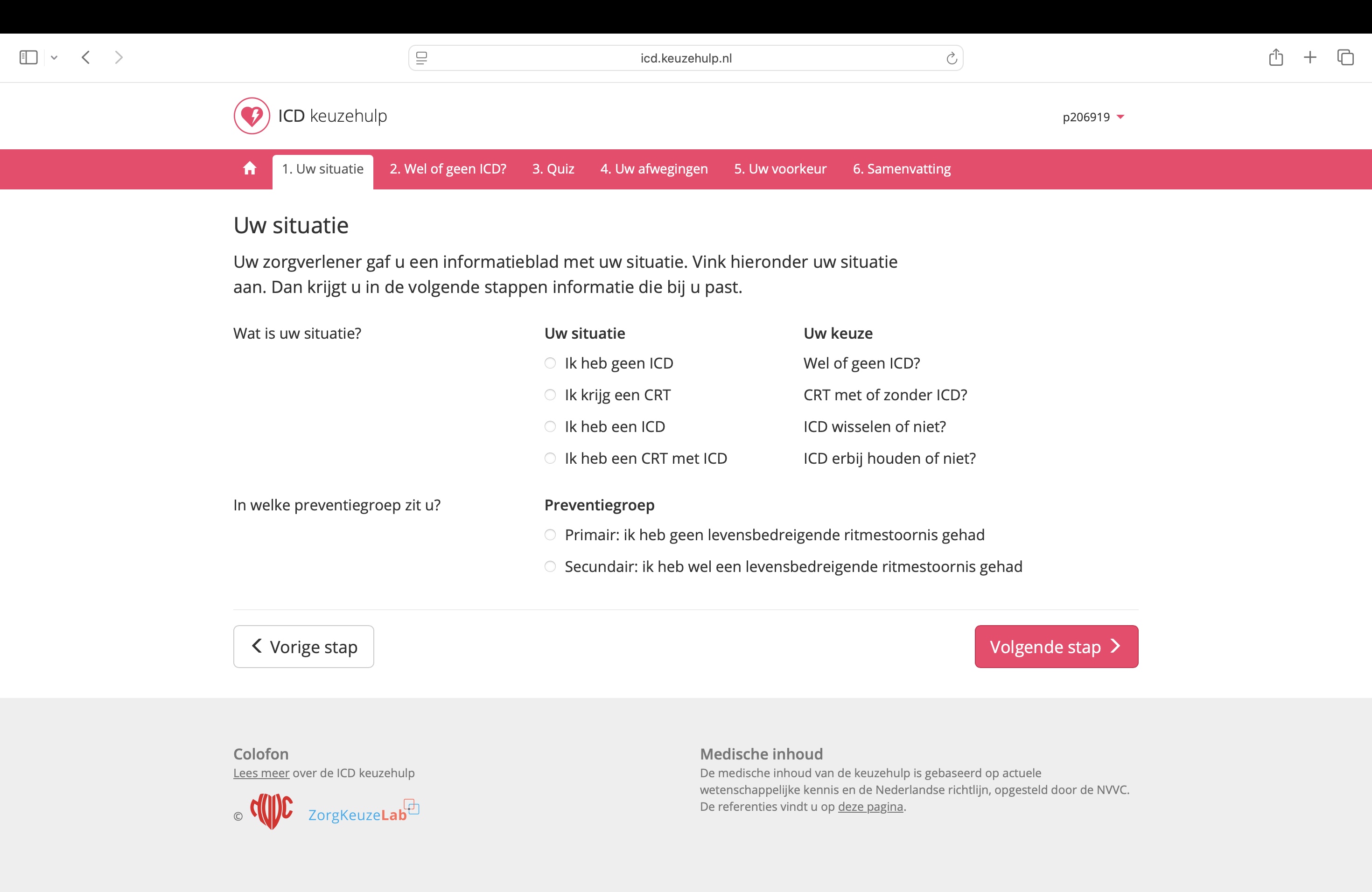


Page for patients to fill out their situation to access specific content:
first device, pulse-generator exchange, type of prevention
